# 593. Population Pharmacokinetic Model Development for Epetraborole and *Mycobacterium avium* Complex (MAC) Lung Disease Patients Using Data from Phase 1 and 2 Studies

**DOI:** 10.1093/ofid/ofac492.645

**Published:** 2022-12-15

**Authors:** Harish Ganesan, M Courtney Safir, Sujata M Bhavnani, Sujata M Bhavnani, Sujata M Bhavnani, Kevin M Krause, Christopher M Rubino

**Affiliations:** Institute for Clinical Pharmacodynamics, Plano, Texas; Institute for Clinical Pharmacodynamics, Plano, Texas; Institute for Clinical Pharmacodynamics, Plano, Texas; Institute for Clinical Pharmacodynamics, Plano, Texas; Institute for Clinical Pharmacodynamics, Plano, Texas; AN2, San Francisco, California; Institute for Clinical Pharmacodynamics, Plano, Texas

## Abstract

**Background:**

Epetraborole (EBO), an orally available bacterial leucyl transfer RNA synthetase inhibitor with potent activity against nontuberculous mycobacteria, is under clinical development for treatment of MAC lung disease. A population pharmacokinetic (PK) model describing the disposition of EBO after oral (PO) and intravenous (IV) administration was developed to support EBO PK-PD analyses and dose selection for patients with MAC lung disease.

**Methods:**

Model development was conducted using NONMEM (v.7.4.3). Models were attempted for oral absorption, systemic compartments, and linear vs. non-linear elimination. Model evaluation involved goodness-of-fit plots and prediction-corrected visual predictive plots, which describe the ability of model-based simulations to capture the observed data. Included were data from 5 Phase 1 (3 IV and 2 PO) and 2 Phase 2 studies (IV only) (**Table 1**).

**Figure:**
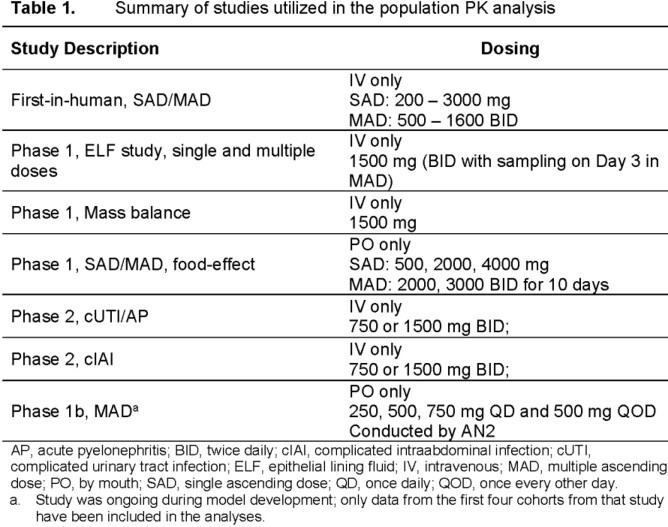

**Results:**

The pooled dataset included 2637 EBO PK samples from 138 subjects/patients. A robust fit to observed data across studies was obtained using a three-compartment model with linear elimination (**Table 2**). The impact of body weight on PK was included using an allometric scaling approach to accommodate observed lower body weight in MAC lung disease patients. PO dosing was modeled using an absolute bioavailability term (F) and transit compartments with separate absorption rates for fed and fasted administration. Interindividual variability (IIV) in systemic clearance was low (7.9%), but IIV in F (32.7%) contributed to slightly higher variability in PK for PO vs. IV administration. Moderate shrinkage was observed for the IIV in the model parameters. This was considered acceptable given inclusion of patients with limited PK data in the model and the objective to facilitate simulations designed to inform dose selection. The prediction-corrected visual predictive check plots for the data obtained from a recently completed Phase 1b study evaluating 28-day oral dosing regimens (NCT04892641) are provided in **Figure 1**.

**Figure:**
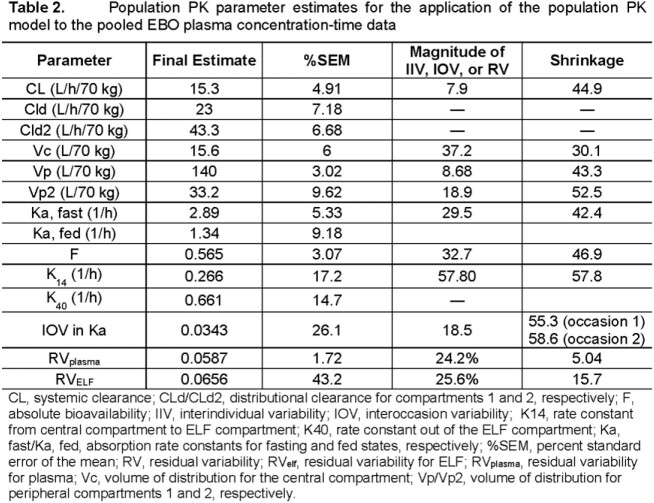

**Figure:**
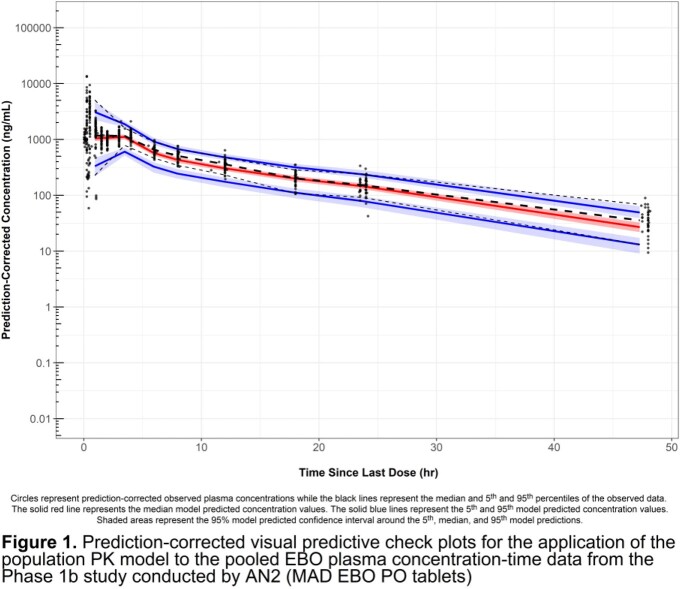

**Conclusion:**

This model is useful for describing expected PK in MAC lung disease patients and was used to conduct simulations for the advancement of the oral EBO 500 mg q24h dosing regimen into clinical studies in patients with MAC lung disease.

**Disclosures:**

**Harish Ganesan, MS**, Adagio Therapeutics, Inc.: Grant/Research Support|Amplyx Pharmaceuticals, Inc.: Grant/Research Support|AN2 Therapeutics: Grant/Research Support|Antabio SAS: Grant/Research Support|Arcutis Biotherapeutics, Inc.: Grant/Research Support|B. Braun Medical Inc.: Grant/Research Support|Basilea Pharmaceutica: Grant/Research Support|Boston Pharmaceuticals: Grant/Research Support|Celdara Medical LLC: Grant/Research Support|Cidara Therapeutics Inc.: Grant/Research Support|Cipla USA: Grant/Research Support|Crestone Inc.: Grant/Research Support|CXC: Grant/Research Support|Debiopharm International SA: Grant/Research Support|Entasis Therapeutics: Grant/Research Support|Evopoint Biosciences Co.: Grant/Research Support|Fedora Pharmaceuticals: Grant/Research Support|GlaxoSmithKline: Grant/Research Support|Hoffmann-La Roche: Grant/Research Support|Insmed Inc.: Grant/Research Support|Iterum Therapeutics Limited: Grant/Research Support|Kaizen Bioscience, Co.: Grant/Research Support|KBP Biosciences USA: Grant/Research Support|Lassen Therapeutics: Grant/Research Support|Matinas Biopharma: Grant/Research Support|Meiji Seika Pharma Co.Meiji Seika Pharma Co., Ltd: Grant/Research Support|Melinta Therapeutics: Grant/Research Support|Menarini Ricerche S.p.A: Grant/Research Support|Mutabilis: Grant/Research Support|Nabriva Therapeutics AG: Grant/Research Support|Novartis Pharmaceuticals Corp.: Grant/Research Support|Paratek Pharmaceuticals, Inc.: Grant/Research Support|PureTech Health: Grant/Research Support|Sfunga Therapeutics: Grant/Research Support|Spero Therapeutics: Grant/Research Support|Suzhou Sinovent Pharmaceuticals Co.: Grant/Research Support|TauRx Therapeutics: Grant/Research Support|Tetraphase Pharmaceuticals: Grant/Research Support|tranScrip Partners: Grant/Research Support|Utility Therapeutics: Grant/Research Support|Valanbio Therapeutics, Inc.: Grant/Research Support|VenatoRx: Grant/Research Support|Wockhardt Bio AG: Grant/Research Support **M. Courtney Safir, PharmD**, Adagio Therapeutics, Inc: Grant/Research Support|Amplyx Pharmaceuticals, Inc.: Grant/Research Support|AN2 Therapeutics: Grant/Research Support|Antabio SAS: Grant/Research Support|Arcutis Biotherapeutics, Inc.: Grant/Research Support|B. Braun Medical Inc.: Grant/Research Support|Basilea Pharmaceutica: Grant/Research Support|Boston Pharmaceuticals: Grant/Research Support|Celdara Medical LLC: Grant/Research Support|Cidara Therapeutics Inc: Grant/Research Support|Cipla USA: Grant/Research Support|Crestone Inc: Grant/Research Support|CXC: Grant/Research Support|Debiopharm International SA: Grant/Research Support|Entasis Therapeutics: Grant/Research Support|Evopoint Biosciences Co: Grant/Research Support|Fedora Pharmaceuticals: Grant/Research Support|GlaxoSmithKline: Grant/Research Support|Hoffmann-La Roche: Grant/Research Support|Insmed Inc.: Grant/Research Support|Iterum Therapeutics Limited,: Grant/Research Support|Kaizen Bioscience, Co.: Grant/Research Support|KBP Biosciences USA: Grant/Research Support|Lassen Therapeutics: Grant/Research Support|Matinas Biopharma: Grant/Research Support|Meiji Seika Pharma Co., Ltd.: Grant/Research Support|Melinta Therapeutics: Grant/Research Support|Menarini Ricerche S.p.A.: Grant/Research Support|Mutabilis: Grant/Research Support|Nabriva Therapeutics AG: Grant/Research Support|Novartis Pharmaceuticals Corp.: Grant/Research Support|Paratek Pharmaceuticals, Inc.: Grant/Research Support|PureTech Health: Grant/Research Support|Sfunga Therapeutics: Grant/Research Support|Spero Therapeutics: Grant/Research Support|Suzhou Sinovent Pharmaceuticals Co.: Grant/Research Support|TauRx Therapeutics: Grant/Research Support|Tetraphase Pharmaceuticals: Grant/Research Support|tranScrip Partners: Grant/Research Support|Utility Therapeutics: Grant/Research Support|Valanbio Therapeutics, Inc.: Grant/Research Support|VenatoRx: Grant/Research Support|Wockhardt Bio AG: Grant/Research Support **Sujata M. Bhavnani, PharmD; MS; FIDSA**, Adagio Therapeutics, Inc: Grant/Research Support|Amplyx Pharmaceuticals, Inc: Grant/Research Support|AN2 Therapeutics: Grant/Research Support|Antabio SAS: Grant/Research Support|Arcutis Biotherapeutics, Inc: Grant/Research Support|B. Braun Medical Inc: Grant/Research Support|Basilea Pharmaceutica: Grant/Research Support|Boston Pharmaceuticals: Grant/Research Support|Bravos Biosciences: Ownership Interest|Celdara Medical LLC: Grant/Research Support|Cidara Therapeutics Inc: Grant/Research Support|Cipla USA: Grant/Research Support|Crestone Inc.: Grant/Research Support|CXC: Grant/Research Support|Debiopharm International SA: Grant/Research Support|Entasis Therapeutics: Grant/Research Support|Evopoint Biosciences Co.: Grant/Research Support|Fedora Pharmaceuticals: Grant/Research Support|GlaxoSmithKline: Grant/Research Support|Hoffmann-La Roche: Grant/Research Support|ICPD: Ownership Interest|ICPD Biosciences, LLC.: Ownership Interest|Insmed Inc: Grant/Research Support|Iterum Therapeutics Limited: Grant/Research Support|Kaizen Bioscience, Co.: Grant/Research Support|KBP Biosciences USA: Grant/Research Support|Lassen Therapeutics: Grant/Research Support|Matinas Biopharma: Grant/Research Support|Meiji Seika Pharma Co., Ltd.: Grant/Research Support|Melinta Therapeutics: Grant/Research Support|Menarini Ricerche S.p.A: Grant/Research Support|Mutabilis: Grant/Research Support|Nabriva Therapeutics AG: Grant/Research Support|Novartis Pharmaceuticals Corp: Grant/Research Support|Paratek Pharmaceuticals, Inc.: Grant/Research Support|PureTech Health: Grant/Research Support|Sfunga Therapeutics: Grant/Research Support|Spero Therapeutics: Grant/Research Support|Suzhou Sinovent Pharmaceuticals Co.: Grant/Research Support|TauRx Therapeutics: Grant/Research Support|Tetraphase Pharmaceuticals: Grant/Research Support|tranScrip Partners: Grant/Research Support|Utility Therapeutics: Grant/Research Support|Valanbio Therapeutics, Inc: Grant/Research Support|VenatoRx: Grant/Research Support|Wockhardt Bio AG: Grant/Research Support **Sujata M. Bhavnani, PharmD; MS; FIDSA**, Adagio Therapeutics, Inc: Grant/Research Support|Amplyx Pharmaceuticals, Inc: Grant/Research Support|AN2 Therapeutics: Grant/Research Support|Antabio SAS: Grant/Research Support|Arcutis Biotherapeutics, Inc: Grant/Research Support|B. Braun Medical Inc: Grant/Research Support|Basilea Pharmaceutica: Grant/Research Support|Boston Pharmaceuticals: Grant/Research Support|Bravos Biosciences: Ownership Interest|Celdara Medical LLC: Grant/Research Support|Cidara Therapeutics Inc: Grant/Research Support|Cipla USA: Grant/Research Support|Crestone Inc.: Grant/Research Support|CXC: Grant/Research Support|Debiopharm International SA: Grant/Research Support|Entasis Therapeutics: Grant/Research Support|Evopoint Biosciences Co.: Grant/Research Support|Fedora Pharmaceuticals: Grant/Research Support|GlaxoSmithKline: Grant/Research Support|Hoffmann-La Roche: Grant/Research Support|ICPD: Ownership Interest|ICPD Biosciences, LLC.: Ownership Interest|Insmed Inc: Grant/Research Support|Iterum Therapeutics Limited: Grant/Research Support|Kaizen Bioscience, Co.: Grant/Research Support|KBP Biosciences USA: Grant/Research Support|Lassen Therapeutics: Grant/Research Support|Matinas Biopharma: Grant/Research Support|Meiji Seika Pharma Co., Ltd.: Grant/Research Support|Melinta Therapeutics: Grant/Research Support|Menarini Ricerche S.p.A: Grant/Research Support|Mutabilis: Grant/Research Support|Nabriva Therapeutics AG: Grant/Research Support|Novartis Pharmaceuticals Corp: Grant/Research Support|Paratek Pharmaceuticals, Inc.: Grant/Research Support|PureTech Health: Grant/Research Support|Sfunga Therapeutics: Grant/Research Support|Spero Therapeutics: Grant/Research Support|Suzhou Sinovent Pharmaceuticals Co.: Grant/Research Support|TauRx Therapeutics: Grant/Research Support|Tetraphase Pharmaceuticals: Grant/Research Support|tranScrip Partners: Grant/Research Support|Utility Therapeutics: Grant/Research Support|Valanbio Therapeutics, Inc: Grant/Research Support|VenatoRx: Grant/Research Support|Wockhardt Bio AG: Grant/Research Support **Sujata M. Bhavnani, PharmD; MS; FIDSA**, Adagio Therapeutics, Inc: Grant/Research Support|Amplyx Pharmaceuticals, Inc: Grant/Research Support|AN2 Therapeutics: Grant/Research Support|Antabio SAS: Grant/Research Support|Arcutis Biotherapeutics, Inc: Grant/Research Support|B. Braun Medical Inc: Grant/Research Support|Basilea Pharmaceutica: Grant/Research Support|Boston Pharmaceuticals: Grant/Research Support|Bravos Biosciences: Ownership Interest|Celdara Medical LLC: Grant/Research Support|Cidara Therapeutics Inc: Grant/Research Support|Cipla USA: Grant/Research Support|Crestone Inc.: Grant/Research Support|CXC: Grant/Research Support|Debiopharm International SA: Grant/Research Support|Entasis Therapeutics: Grant/Research Support|Evopoint Biosciences Co.: Grant/Research Support|Fedora Pharmaceuticals: Grant/Research Support|GlaxoSmithKline: Grant/Research Support|Hoffmann-La Roche: Grant/Research Support|ICPD: Ownership Interest|ICPD Biosciences, LLC.: Ownership Interest|Insmed Inc: Grant/Research Support|Iterum Therapeutics Limited: Grant/Research Support|Kaizen Bioscience, Co.: Grant/Research Support|KBP Biosciences USA: Grant/Research Support|Lassen Therapeutics: Grant/Research Support|Matinas Biopharma: Grant/Research Support|Meiji Seika Pharma Co., Ltd.: Grant/Research Support|Melinta Therapeutics: Grant/Research Support|Menarini Ricerche S.p.A: Grant/Research Support|Mutabilis: Grant/Research Support|Nabriva Therapeutics AG: Grant/Research Support|Novartis Pharmaceuticals Corp: Grant/Research Support|Paratek Pharmaceuticals, Inc.: Grant/Research Support|PureTech Health: Grant/Research Support|Sfunga Therapeutics: Grant/Research Support|Spero Therapeutics: Grant/Research Support|Suzhou Sinovent Pharmaceuticals Co.: Grant/Research Support|TauRx Therapeutics: Grant/Research Support|Tetraphase Pharmaceuticals: Grant/Research Support|tranScrip Partners: Grant/Research Support|Utility Therapeutics: Grant/Research Support|Valanbio Therapeutics, Inc: Grant/Research Support|VenatoRx: Grant/Research Support|Wockhardt Bio AG: Grant/Research Support **Kevin M. Krause, MBA**, AN2 Therapeutics: Employee|AN2 Therapeutics: Stocks/Bonds **Christopher M. Rubino, PharmD**, Adagio Therapeutics: Grant/Research Support|Amplyx Pharmaceuticals, Inc: Grant/Research Support|AN2 Therapeutics: Grant/Research Support|Antabio SAS: Grant/Research Support|Arcutis Biotherapeutics, Inc: Grant/Research Support|B. Braun Medical Inc.: Grant/Research Support|Basilea Pharmaceutica: Grant/Research Support|Boston Pharmaceuticals: Grant/Research Support|Bravos Biosciences: Ownership Interest|Celdara Medical LLC: Grant/Research Support|Cidara Therapeutics Inc: Grant/Research Support|Cipla USA: Grant/Research Support|Crestone Inc: Grant/Research Support|CXC: Grant/Research Support|Debiopharm International SA: Grant/Research Support|Entasis Therapeutics: Grant/Research Support|Evopoint Biosciences Co.: Grant/Research Support|Fedora Pharmaceuticals: Grant/Research Support|GlaxoSmithKline: Grant/Research Support|Hoffmann-La Roche: Grant/Research Support|ICPD: Ownership Interest|ICPD Biosciences, LLC.: Ownership Interest|Insmed Inc.: Grant/Research Support|Iterum Therapeutics Limited: Grant/Research Support|Kaizen Bioscience, Co.: Grant/Research Support|KBP Biosciences USA: Grant/Research Support|Lassen Therapeutics: Grant/Research Support|Matinas Biopharma: Grant/Research Support|Meiji Seika Pharma Co., Ltd.: Grant/Research Support|Melinta Therapeutics: Grant/Research Support|Menarini Ricerche S.p.A: Grant/Research Support|Mutabilis: Grant/Research Support|Nabriva Therapeutics AG: Grant/Research Support|Novartis Pharmaceuticals Corp.: Grant/Research Support|Paratek Pharmaceuticals, Inc.: Grant/Research Support|PureTech Health: Grant/Research Support|Sfunga Therapeutics: Grant/Research Support|Spero Therapeutics,: Grant/Research Support|Suzhou Sinovent Pharmaceuticals Co.: Grant/Research Support|TauRx Therapeutics: Grant/Research Support|Tetraphase Pharmaceuticals: Grant/Research Support|tranScrip Partners: Grant/Research Support|Utility Therapeutics: Grant/Research Support|Valanbio Therapeutics, Inc.: Grant/Research Support|VenatoRx: Grant/Research Support|Wockhardt Bio AG: Grant/Research Support.

